# Depth of Invasion of 13 mm or Greater Accurately Predicted the Risk of Having a Node Positivity in Lymphadenectomy for Squamous Vulvar Cancer

**DOI:** 10.1111/jog.70215

**Published:** 2026-02-20

**Authors:** Rafael Coelho Albuquerque, Samanta Santos Sousa, Gabriel Valente Tozatti, Beatriz Boneto, Jose Carlos Torres, Diama Bhadra Vale

**Affiliations:** ^1^ Department of Obstetrics and Gynecology University of Campinas Campinas Brazil

**Keywords:** lymph node excision, prognosis, sentinel lymph node biopsy, vulvar neoplasm, vulvectomy

## Abstract

**Aim:**

To analyze factors related to lymph node involvement in patients with squamous vulvar cancer undergoing lymphadenectomy and wide local excision at the University Hospital in Campinas, Brazil.

**Methods:**

A retrospective study involving 56 women treated between 2010 and 2022. The primary outcome was inguinal lymph node involvement (positive or negative). Clinical, operative, and pathologic variables were analyzed by appropriate tests. Kaplan–Meier curves were used to determine overall survival rate (OS). A receiver operating characteristic (ROC) curve was created to determine the optimal value of the depth of invasion for predicting node positivity.

**Results:**

Of the 56 women who underwent surgery, node involvement was positive in 18 (32.1%). Where node was positive, the tumors were over 5 cm in 22.2% (versus 2.7%, *p* = 0.035), had an depth of invasion equal to or deeper than 13 mm (13 mm+) in 62.5% (versus 14.3%, *p* < 0.001), lymphovascular invasion in 33.3% (versus 11.8%, *p* = 0.024), presented disease progression in 44.4% (versus 11.8%, *p* = 0.019), and death in 77.8% (versus 31.6%, *p* = 0.001). The 5‐year OS was 57.1% in the node‐negative and 8.6% in the node‐positive group, with most events occurring within the first 24 months. Depth of invasion 13 mm + increased the risk of node‐positivity 11 times (11.37;1.85–69.82), showing a predictive negative value of 83.3%, and accuracy of 78.4%.

**Conclusion:**

The 13.0 mm cutoff for depth of invasion was independently associated with the risk of having a positive node, with an accuracy of 78.4%. The 5‐year OS was 8.6% in the node‐positive group, with most events within the first 24 months.

## 1 Introduction

The main prognostic factor for vulvar cancer is lymph node status [1, 2, 3, 4]. Women have more favorable disease‐free survival and overall mortality rates when the diagnosis is made at an early stage [5, 6]. The treatment modality of choice is surgery as it is associated with better outcomes and should be indicated whenever feasible [7, 8, 9]. Where surgery is not possible, radiotherapy is preferable, either alone or in combination with chemotherapy [5]. Therefore, surgical evaluation of lymph node status is a cornerstone in establishing the correct staging and prognosis for the patient [10].

An essential characteristic of patients with vulvar cancer is their advanced age [5, 11, 12]. This presents a challenge for determining the correct course of action, as the association with comorbidities and physiological aspects related to senility can make it difficult to determine the best management approach to achieve the best oncological outcomes. The evaluation of factors associated with the diagnosis of affected lymph nodes can assist in the clinical and surgical management of these women.

This study aimed to analyze the clinical, surgical, and pathological factors related to the presence of lymph node involvement in patients undergoing lymphadenectomy associated with the wide local excision of vulvar cancer, admitted for primary treatment at a reference center dedicated to women's health.

## 2 Methods

This is a retrospective longitudinal observational study conducted through the analysis of medical records of women with a confirmed vulvar cancer diagnosis who underwent primary treatment at the Women's Hospital of the University of Campinas (CAISM/Unicamp), in Campinas, in the state of São Paulo, Brazil.

Women were referred to the Women's Hospital by primary care or referral clinics from the public health system in the region of the university. The total population served by the Women's Hospital is approximately three million people, primarily residing in urban areas. During the first visit, a physical examination was performed in the presence of a senior oncology gynecologist. Patients were staged according to the FIGO 2013 guidelines, assisted by imaging tests available at the center (ultrasound, computed tomography, and/or magnetic resonance imaging). After surgery, the women returned weekly for clinical evaluation of the surgical wound until it was in the final phase of healing. Within 30 to 45 days of the surgery, the anatomopathological result was reviewed to determine whether adjuvant therapy should be performed. Any radiotherapy or chemotherapy required was also administered at the Women's Hospital. After treatment, women were followed up every 4–6 months for at least 5 years. Imaging was not routinely performed during follow‐up unless an abnormality was clinically detected.

The study subjects were all women with a diagnosis of vulvar cancer admitted to the unit between 2010 and 2022 who underwent surgery involving the approach of inguinal lymph nodes as the primary treatment and whose pathological final diagnosis was squamous cell carcinoma. The total sample consisted of 56 women, of whom eight underwent sentinel lymph node biopsy (SLNB) as the primary approach to the inguinal lymph nodes. In one case of SLNB, cancer cells were identified with a frozen section biopsy, and the woman underwent a lymphadenectomy at the same surgery time. No overstaging was observed in the seven remaining women. In 48 women, the lymphadenectomy was the primary approach, either being uni‐ or bilateral, according to the laterality of the tumor.

The primary outcome of this study was the presence of lymph node involvement, categorized as either positive or negative. The other variables included age, current smoking status, pathological staging, largest diameter of the excised tumor, depth of invasion, presence of angiolymphatic invasion, association with Vulvar Intraepithelial Neoplasia (VIN), length of surgery, recurrence or disease progression, and overall survival. The cutoff value of 5 cm for the largest diameter was based on the distribution of internal data; the depth of invasion was measured from the epithelial‐stromal junction of the most superficial dermal papilla, located adjacent to the deepest point of tumor invasion.

The statistical analysis employed a chi‐square test, Fisher's exact test, or a Mann–Whitney test. For overall survival (OS), the initial time (T0) was defined as the date of surgery, and the outcomes were death (date of death) and censoring (date of the last recorded consultation for patients considered “alive”). Kaplan–Meier curves, along with log‐rank tests, were used. Simple and multiple logistic regression analyses were performed to determine the risk associated with node status. All significant variables (*p* < 0.05) in the univariate analysis were included in the multivariate analysis. A receiver operating characteristic (ROC) curve was created to determine the optimal value of the depth of invasion to predict a positive lymph node. The significance level adopted for all statistical tests was 5% (*p* < 0.05).

The Research Ethics Committee (CEP) of the University of Campinas approved the study under number 45223421.0.0000.5404. The need for informed consent from patients was waived due to the study's retrospective nature.

## 3 Results

Overall, of the 56 women who underwent surgery, 38 (67.9%) had no cancer cells observed in their lymph nodes (node‐negative). In contrast, in 18 cases (32.1%), metastasis was observed in at least one lymph node (node‐positive). Regarding the pathological variables, in the node‐positive group, tumors were more frequently over 5 cm in diameter (*p* = 0.035) and had a depth of invasion equal to or greater than 13 mm (*p* < 0.001). In this node‐positive group, more lymphovascular invasion (*p* = 0.024) and association with VIN (*p* = 0.020) were observed. Regarding any follow‐up characteristics, node‐positive women presented more frequently with lymphedema (*p* = 0.060), disease progression (*p* = 0.019), and death (*p* = 0.001) compared to node‐negative women. Being a smoker was not associated with node status (Table 1).

**TABLE 1:** Clinical and pathological variables of 56 women who underwent surgery for vulvar cancer treatment with inguinal lymphadenectomy by node status.

	Node negative	Node positive	Total	*p*
Current smoker				
No	34 (89.5)	12 (66.7)	46 (82.2)	0.060
Yes	04 (10.5)	06 (33.3)	10 (17.8)	
Greater tumor diameter				
≤ 5 cm	36 (97.3)	14 (77.8)	50 (90.9)	0.035
> 5 cm	1 (2.7)	4 (22.2)	5 (9.1)	
Depth of invasion^a^				
< 13 mm	30 (85.7)	6 (37.5)	36 (70.5)	< 0.001
≥ 13 mm	5 (14.3)	10 (62.5)	15 (29.5)	
Lymphovascular invasion				
No	35 (92.1)	12 (66.7)	47 (83.9)	0.024
Yes	3 (7.9)	6 (33.3)	9 (16.1)	
VIN association				
No	17 (44.7)	14 (77.8)	31 (55.4)	0.020
Yes	21 (55.3)	4 (22.2)	25 (44.6)	
Lymphedema				
No	34 (89.5)	12 (66.7)	46 (82.1)	0.060
Yes	4 (10.5)	6 (33.3)	10 (17.9)	
Relapse				
No	27 (79.4)	7 (38.9)	34 (60.7)	0.019
Yes	7 (20.6)	3 (16.7)	10 (17.9)	
Disease progression	4 (11.8)	8 (44.4)	12 (21.4)	
Mortality				
No	26 (68.4)	4 (22.2)	30 (53.6)	0.001
Yes	12 (31.6)	14 (77.8)	26 (46.4)	

Abbreviation: VIN, vulvar intraepithelial neoplasia.

^a^ Data not available for five cases. In the 15 cases in which the depth of invasion was 13 mm or more, in nine cases, the overall diameter was lower than 4 cm (five of them were node positive).

Among the perioperative characteristics of the groups, no differences were observed in total surgery time, total hospital stay length, or the number of nodes removed. However, even if it was not statistically significant, the number of days spent in the hospital was almost twice as high in the node‐positive group, and a slightly higher number of nodes removed was observed with node‐positive women (Table 2).

**TABLE 2:** Operatory variables of 56 women who underwent surgery for vulvar cancer treatment with inguinal lymphadenectomy by node status.

	Average	ST	Median	*p*
Total surgery time (minutes)				
Node negative	164.4	70.6	150.0	0.257
Node positive	170.0	45.0	165.0	
Total hospital stay (days)				
Node negative	3.3	1.8	3.0	0.091
Node positive	6.5	6.4	3.0	
Number of nodes removed				
Node negative	11.8	7.5	11.5	0.165
Node positive	14.8	7.5	16.0	

Abbreviation: ST, standard deviation.

**FIGURE 1:**
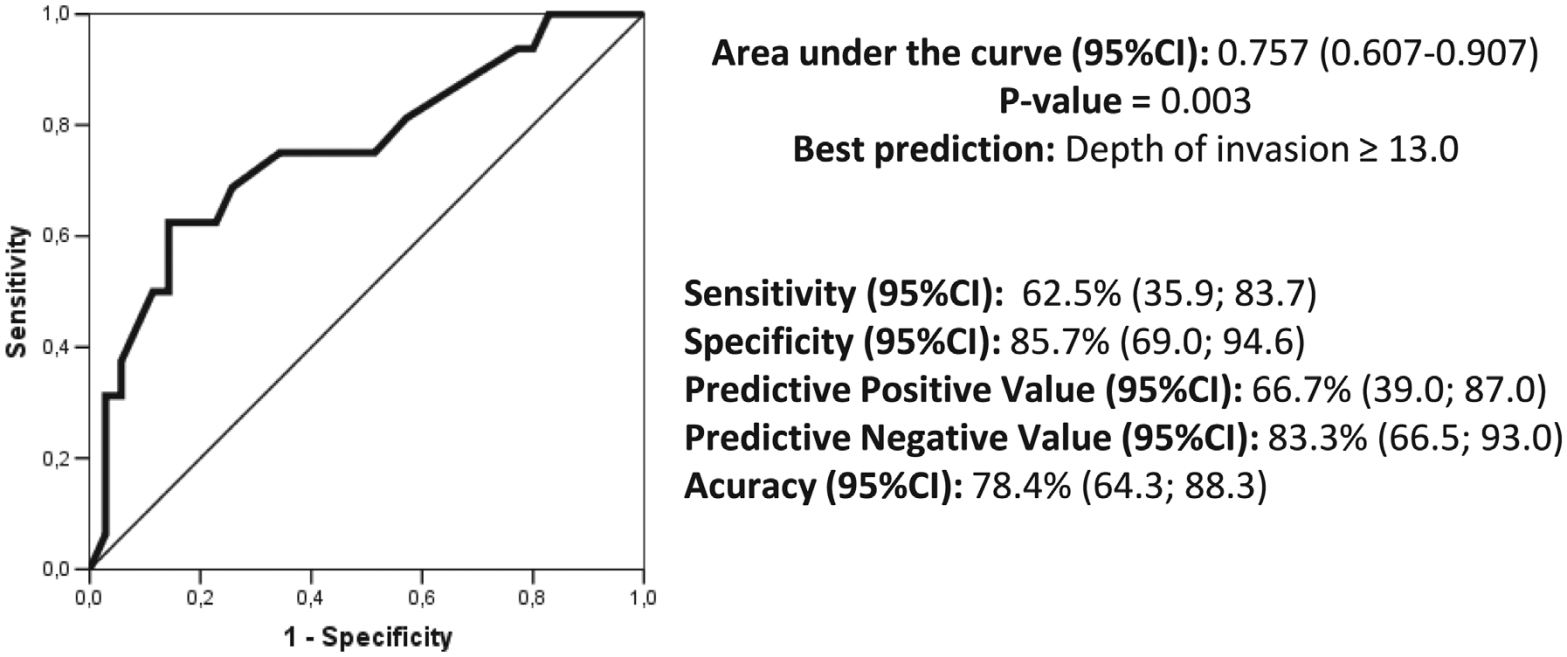
Cumulative overall survival of 56 women who underwent surgery for vulvar cancer treatment with inguinal lymphadenectomy by node status.

Figure 1 shows the Kaplan–Meier curves of overall survival by node status. Node‐positive women had significantly lower survival rates compared to node‐negative women (*p* < 0.001). In node‐negative women, the overall 2‐year survival rate was 82.9%, decreasing to 57.1% after 5 years. In the node‐positive women, most of the deaths occurred in the first 24 months (2‐year overall survival of 43.2%) with the final deaths occurring after 48 months.

Next, the variables were examined to identify the risk of having a node‐positive result (Table 3). Being a current smoker, having a tumor bigger than 5 cm at the greatest diameter, observing a depth of invasion equal to or over 13 mm, lymphovascular invasion, and VIN association significantly increased the risk of a node‐positive. However, in the multivariate analysis, only depth of invasion remained independently related; women whose tumor presented a depth of invasion equal to or greater than 13 mm had 11 times the risk of being node‐positive than women with a depth of invasion lower than 13 mm.

**TABLE 3:** Associated factors with the risk of having a node‐positive in 56 women who underwent surgery for vulvar cancer treatment with inguinal lymphadenectomy.

	Simple regression			Multiple regression		
	*p*	OR	95% CI	*p*	OR	95% CI
Current smoker						
No	—	1.00	—	—	1.00	—
Yes	0.047	4.25	1.02–17.69	0.105	5.33	0.71–40.18
Tumor diameter						
≤ 5 cm	—	1.00	—	—	1.00	—
> 5 cm	0.045	10.28	1.06–100.14	0.841	0.73	0.04–15.23
Depth of invasion						
< 13 mm	—	1.00	—	—	1.00	—
≥ 13 mm	0.001	10.00	2.50–39.98	0.009	11.37	1.85–69.82
LV invasion						
No	—	1.00	—	—	1.00	—
Yes	0.024	5.83	1.26–27.03	0.145	5.06	0.57–44.75
VIN association						
No	—	1.00	—	—	1.00	—
Yes	0.025	4.32	1.20–15.58	0.267	2.38	0.52–10.97

Abbreviations: CI, confidence interval; LV, lymphovascular; OR, odds ratio; *p*, *p*‐value; VIN, vulvar intraepithelial neoplasia.

**FIGURE 2:**
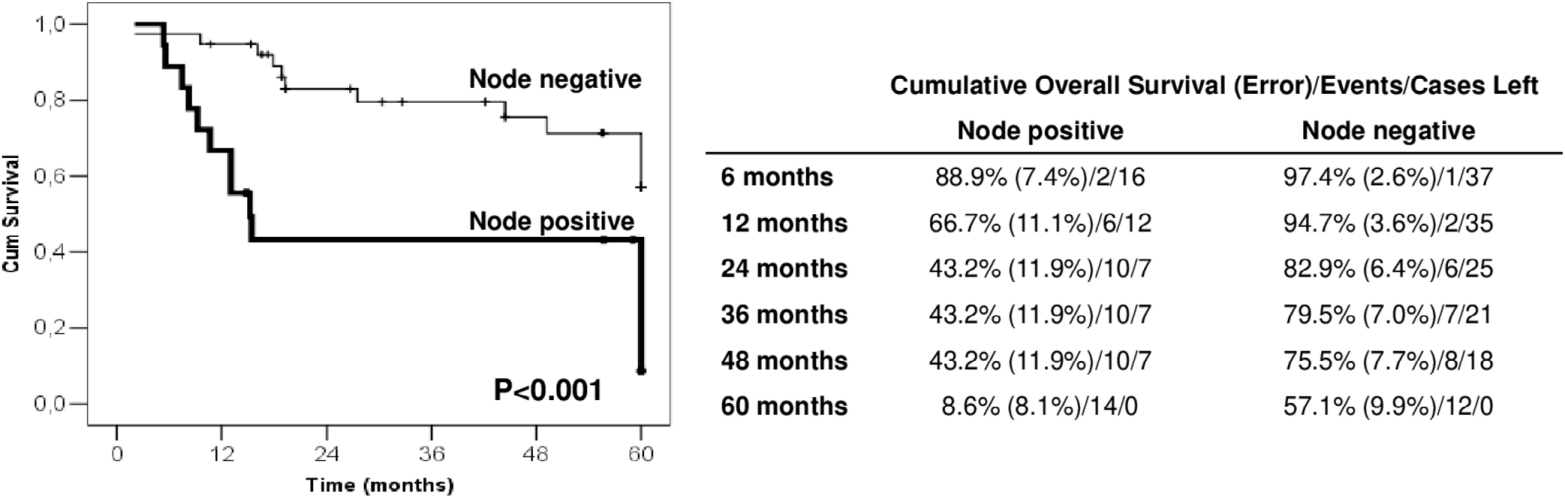
Receiver‐operating characteristic curve for the best prediction of the depth of invasion of being node‐positive in 56 women who underwent surgery for vulvar cancer treatment with inguinal lymphadenectomy. CI, confidence interval.

A ROC curve was plotted to define the best prediction of the depth of invasion associated with a node‐positive outcome. This showed the 13.0 mm cutoff being the best cutoff value (Figure 2). In this situation, the predictive negative value was 83.3%, and the accuracy was 78.4%.

## 4 Discussion

In this study, we found 32.1% of women who underwent surgery for vulvar cancer treatment with inguinal lymphadenectomy were positive for lymph node metastasis. Here, the only independent variable for being node‐positive for cancer cells was the depth of invasion, with a cutoff point equal to or greater than 13 mm.

Lymph node status is the most important prognostic factor in vulvar cancer [13, 14]. Our value of node‐positive women was almost identical to that observed in the classic Groningen International Study on Sentinel nodes in Vulvar cancer (GROINSS‐V) study (32.9%) [15].

Depth of invasion is already recognized as a predictor of recurrence where a cutoff value of 1 mm determines the stage and the need for groin surgery [1, 5, 16, 17]. However, the prediction for presenting lymph node metastasis is not well explored in women who undergo inguinal lymph node dissection. In an ROC analysis, we found the 13.0 mm cutoff to be the best value for predicting node status. Node metastasis was 11 times more likely to occur where the tumor presented a depth of invasion equal to or over 13 mm. In surgical management, it might be harmful to indicate SLN if there is a previous indication of profound invasion depth.

In our sample, in nine out of the 15 cases where the depth of invasion was 13 mm or more, the greater tumor diameter was lower than 4 cm. The size of the primary tumor is less relevant in determining the prognosis. However, it has previously been associated with lymph node metastasis [2, 6, 18, 19, 20]. In the GROINSS‐V study, the cutoff of 4 cm was chosen to indicate SLN or not [18], but this is not consistent over the current literature [2, 6, 19, 20]. In fact, in our study, a 5 cm cutoff was found to be significant in a univariate analysis. Aragona et al. suggest that larger tumors, extra‐nodal growth, and the number of positive lymph nodes may indicate poorer survival, with these patients requiring a tailored treatment [19].

In our univariate analysis, we found that lymphovascular invasion was a predictor of node metastasis. Although it is not a criterion for indicating whether an SLN is present or not, it should also be considered a high‐risk factor when evaluating a negative SLN. The role of vulvar intraepithelial neoplasia (VIN) was also highlighted as when associated with invasive disease. VIN may indicate earlier lesions, tending to restrict involvement to the local region and a higher proportion of negative lymph nodes.

Women with negative nodes had half as long hospital stays compared to node‐positive women. The hypothesis is that in this group, which included the women who biopsied an SLN without having a lymphadenectomy, had a more conservative procedure with a reduced morbidity. Clinical lymphedema was observed in 17.9% of women during follow‐up, which was more frequent than in those with node metastasis. A systematic review and meta‐analysis of vulvar cancer observed a lymphedema incidence of 32.1% after systematic lymphadenectomy and 5.9% after SLN [21]. The method used to assess the diagnosis may have influenced the incidence data utilized. Only one study included in this review evaluated node status after lymphadenectomy and found no relation between the number of total positive nodes and long‐term lymphedema [22].

As expected, in the node‐positive group, we found more frequent disease progression and more deaths. In node‐negative women, the overall 2‐year survival rate was 82.9%, decreasing to 57.1% after 5 years. In the node‐positive women, the 2‐year overall survival rate was 43.2%. Any further deaths only occurred after 48 months. In the American SEER survival analysis, among women presenting with no lymph node involvement (stages I and II), the overall 5‐year survival rate was 60%. When the lymph node was involved (stage III), the 5‐year survival rate was approximately 28%, and at stage IV, the survival rate dropped to 6% [7]. In clinical practice, 2 years is an essential marker in the course of the disease.

The strength of this study lies in its sample size, given that this is a rare neoplasm. Furthermore, obtaining qualified data from a single center is a challenging task. However, the retrospective observational nature of the study reduces the strength of validating the hypotheses presented. In addition, the biological aspects of the tumor were not studied. Future studies are needed to identify the women at the highest risk more accurately. Surgery for vulvar cancer is related to many morbidities but is still the main therapeutic option; developing target therapies based on careful analysis of prognostic variables can improve outcomes.

In conclusion, the 13.0 mm cutoff for depth of invasion was the best value found to predict node positivity, with a predictive negative value of 83.3% and an accuracy of 78.4%. This cutoff for depth of invasion was the only variable independently associated with the risk of having at least one positive node. The 5‐year OS was 8.6% in the node‐positive group, with most events occurring within the first 24 months.

## Author Contributions

**Rafael Coelho Albuquerque:** conceptualization, data curation, formal analysis, investigation, methodology, visualization, writing – review and editing. **Samanta Santos Sousa:** data curation, investigation, methodology, visualization, writing – review and editing. **Gabriel Valente Tozatti:** data curation, investigation, writing – review and editing. **Beatriz Boneto:** data curation, writing – review and editing. **Jose Carlos Torres:** investigation, validation, writing – review and editing. **Diama Bhadra Vale:** conceptualization, data curation, formal analysis, methodology, project administration, supervision, validation, visualization, writing – review and editing.

## Disclosure

The authors have nothing to report.

## Ethics Statement

The Research Ethics Committee of the University of Campinas approved the study under number 45223421.0.0000.5404.

## Consent

The need for informed consent from patients was waived by the Ethics Committee due to the study's retrospective nature.

## Conflicts of Interest

The authors declare no conflicts of interest.

## Data Availability

Data openly available in the public repository https://redu.unicamp.br/ with the doi:10.25824/redu/ZXBHAY.
